# Evidence Around the Impact of Pulmonary Rehabilitation and Exercise on Redox Status in COPD: A Systematic Review

**DOI:** 10.3389/fspor.2021.782590

**Published:** 2021-11-26

**Authors:** Alastair Watson, Tom M. A. Wilkinson, Anna Freeman

**Affiliations:** ^1^Clinical and Experimental Sciences, Faculty of Medicine, University of Southampton, Southampton, United Kingdom; ^2^National Institute for Health Research Southampton Biomedical Research Centre, University Hospital Southampton NHS Foundation Trust, Southampton, United Kingdom; ^3^College of Medical and Dental Sciences, University of Birmingham, Birmingham, United Kingdom

**Keywords:** COPD, pulmonary rehabilitation, exercise, redox status, oxidative stress, systematic review

## Abstract

**Introduction:** Oxidative stress is increasingly recognized as a significant factor in the pathogenesis of chronic obstructive pulmonary disease (COPD). Pulmonary rehabilitation, a major component of which is prescribed exercise, is essential in COPD care. Regular exercise has been proposed to increase antioxidant defenses and overall enhance the ability of the body to counteract oxidative stress. However, the mechanisms through which it improves COPD outcomes remain unclear.

**Objectives:** We aimed to appraise the current evidence around the impact of pulmonary rehabilitation on redox status, compared with other exercise interventions, to gain an understanding of optimal exercise interventions to modify this pathophysiological mechanism.

**Methods:** We performed a systematic review through searching CENTRAL, MEDLINE, PubMed, Scopus, and Web of Science. Results were independently reviewed and relevant studies were selected by two independent assessors. Studies were assessed by two independent people using the modified RoB 2 tool and discrepancies were resolved through discussion.

**Results:** We identified 1,710 records and 1,117 records after duplicate removal. Six studies were included in the final analysis. The evidence available was low quality and four studies had high risk of bias and two studies had unclear risk of bias. Studies were small (15–56 participants); only two included details of randomization and patient cohorts were of varying ages and poorly described. Differences in smoking status and previous exercise levels, which are known to impact redox status, were not well documented. Studies were not standardized and used different exercise doses and measured different outcomes. One study reported lower malondialdehyde levels, a marker of lipid peroxidation, after pulmonary rehabilitation, compared with control. However, one study saw no difference following whole-body vibration training and another study showed higher malondialdehyde levels following supervised modified arm swing exercise compared with control.

**Conclusion:** Understanding the impact of exercise on oxidative stress in COPD could lead to tailored exercise programs and modification of pathological mechanisms. However, we identify a lack of high-quality evidence to determine this. Larger, standardized, and high quality randomized controlled trials (RCTs) are essential, which use carefully clinically characterized and controlled cohorts to determine the relative impact of different exercise interventions on redox status to guide COPD management. We propose an idealized RCT design, which could be used to try and meet this need.

## Introduction

Pulmonary rehabilitation (PR) is one of the most cost-effective interventions in chronic obstructive pulmonary disease (COPD), with demonstration of clinically significant improvements in dyspnea, fatigue, and quality of life (Steiner and Roberts, 2016; Bourne et al., 2017). Specifically, a meta-analysis concluded that PR, with at least 4 weeks of exercise intervention, results in statistically and clinically significant gains in health-related quality of life and functional exercise capacity and it reduces hospital readmissions if undertaken within 1 month of discharge from hospital following acute exacerbation (COPD, 2012). PR guidance does not specify the duration and intensity of exercise recommended and a recent Cochrane review suggests that further research studies should focus on identification of ideal length and intensity of training (Donaldson et al., 2005; McCarthy et al., 2015). Current definitions, as per the 2013 guidelines in an American Thoracic Society/European Respiratory Society statement (Spruit et al., 2013), are only that PR comprises “a comprehensive intervention based on thorough patient assessment followed by patient-tailored therapies that include, but are not limited to, exercise training, education, and self-management intervention aiming at behavior change, designed to improve the physical and psychological condition of people with chronic respiratory disease, and to promote the long-term adherence to health-enhancing behaviors.” This definition of “standard” PR does not allow for a comparable “dose” of exercise to be given with PR and complicates comparisons between PR and other exercise interventions. However, a subsequent systematic review and meta-analysis concluded that high-intensity interval training appeared to be comparable to continuous training with regards to cardiovascular and functional gains (Adolfo et al., 2019). While this provides reassurance that functional gains are less “dose” related, the subtler, metabolomic responses to varying exercise regimes remain unknown. The mechanisms through which exercise confers benefit for patients with COPD remain unclear and are likely multifactorial, but there is emerging evidence that some of the improvements are conveyed through reduction in oxidative stress (Alcazar et al., 2019), with oxidative stress recognized as a major factor in the pathogenesis of COPD (Kirkham and Barnes, 2013; Page et al., 2021). Tobacco smoke is an additional source of exogenous reactive oxygen species (ROS), alongside exposure to pollution and microbial sources. ROS has been implicated in development of smoke-induced damage through multiple mechanisms, including activation of redox-sensitive transcription factors such as nuclear factor-kappa B (NF-κB) and mitogen-activated protein kinases (MAPKs), triggering downstream inflammatory responses, indirect or direct histone modification with induction of inflammatory mediators, and and having an impact on the glucocorticoid receptor activation pathway (Chung and Adcock, 2008). Even after smoking cessation, ROS exposure continues to be driven by both the exogenous factors, such as biomass smoke and air pollution, but additionally increased endogenous ROS and reduced endogenous antioxidant availability as sequelae from COPD-related disease processes in the form of inflammation and infection (Barnes, 2020). Exercise acts as an oxidative stressor, triggering redox-sensitive signaling responses (Webb et al., 2017), with the redox responses to exercise exhibiting wide variability between individuals (Cumpstey et al., 2019). Regular exercise has been proposed to increase the antioxidant defenses of the body and provide an overall increase in the ability to counteract oxidative stress (Done and Traustadóttir, 2016; Freeman et al., 2020). However, the comparable impact of PR and different exercise interventions on redox status in COPD have not been clearly described. We, therefore, undertook a systematic review with an aim of understanding the impact of different intensities and types of exercise intervention on redox status in COPD to gain additional understanding about the optimal exercise-based treatment strategy for modifying this pathophysiological mechanism.

## Methods

### Search Strategy and Selection Criteria

Our research question was: “How does pulmonary rehabilitation or other exercise interventions alter redox status in patients with COPD?” and our Population, Intervention, Comparison, and Outcome (PICO) framework is given in Table 1. We searched the literature to identify interventional trials, which compared patients with COPD (population) who received either pulmonary rehabilitation or another exercise intervention (intervention) vs. a control COPD group with either no exercise intervention or an alternative exercise intervention (comparator). We looked for studies including redox status measures (outcomes). Our inclusion and exclusion criteria are given in Table 2. No restriction was placed on date of article publication. Therefore, articles published up to the search date of 7th July, 2021 were included. Articles were not limited based on language.

**Table 1:** PICO framework.

**Population**	**Adults diagnosed with COPD**
Intervention	Pulmonary rehabilitation or exercise intervention
Comparator	Adults diagnosed with COPD not receiving pulmonary rehabilitation or an alternative exercise intervention
Outcome	Primary outcome: measurement of redox status/oxidative stress

**Table 2:** Inclusion and exclusion criteria.

**Inclusion criteria**	**Exclusion criteria**
1. Peer reviewed primary research	1. Unable to obtain full text (if this occurred, authors were first contacted before exclusion)
2. Humans with COPD	2. Study interventions included a drug/nutritional supplement
3.1 or more exercise or PR intervention	3. Exercise or PR intervention was not included as an intervention
4. Outcome measured related to redox status or oxidative stress	5. The outcomes did not include a measure of redox status
5. Appropriate control group of COPD patients	6. Study was not related to humans with COPD
	7. No appropriate control group of COPD patients

We searched the following databases CENTRAL (the Cochrane Library), MEDLINE (Ovid), PubMed, Scopus, and Web of Science for articles using the relevant search terms (Table 3). We subsequently searched using Google Scholar with the search terms: “COPD” or “Chronic Obstructive Pulmonary Disease” and “Redox” or “oxidative^*^” and “Exercise” or “pulmonary rehabilitation^*^.” The first 1,000 articles (ordered by relevance) were chosen to review the title and abstract. This was pragmatically chosen as articles appearing after article number 500 were deemed highly irrelevant. Reference lists for all the articles included in this study were checked for relevant studies, which may have been missed. Appropriate studies, as determined by title and abstract, were read in full to determine whether to be included or not. Search terms and the search strategy were developed by AW and AF and search results were reviewed independently by AW and AF. Data extraction was carried out by AW and AF, which was cross-validated and any conflicts with respect to inclusion of studies were resolved through discussion. The search strategy and methodology for evidence selection and critical appraisal were established prior to undertaking this systematic review. However, no formal protocol was written and this systematic review was not registered, for example, on The International Prospective Register of Systematic Reviews (PROSPERO) (National Institute for Health Research, 2021).

**Table 3:** Search strategy.

	**Search term**
**1**	“COPD” OR “Chronic Obstructive Pulmonary Disease” OR “obstructive” OR “chronic obstructive airway disease” OR “COAD” OR “chronic obstructive lung disease” OR “COLD” OR “emphysema” OR “chronic bronchitis”
**AND**	
**2**	“Redox” OR “oxidative*”
**AND**	
**3**	“Exercise” OR “pulmonary rehab*”

### Evidence Selection

Studies were selected by two independent reviewers based on the inclusion and exclusion criteria outlined in Table 2 through looking at the title and abstract. Non-relevant articles were excluded and those remaining were subsequently reviewed in full and relevant articles were included. Any discrepancies were resolved by consensus and a third independent reviewer was not required. For articles where the full texts were inaccessible, first and corresponding authors were contacted to gain access and the articles were subsequently included if responses were received.

### Critical Appraisal

All the studies were appraised by using a modified version of the RoB 2 score (Supplementary Table 1) (Cochrane Methods Bias, 2020). Studies were appraised by two independent assessors and any inconsistencies with scoring were resolved through discussion.

## Results

### Search Results

**Figure 1:**
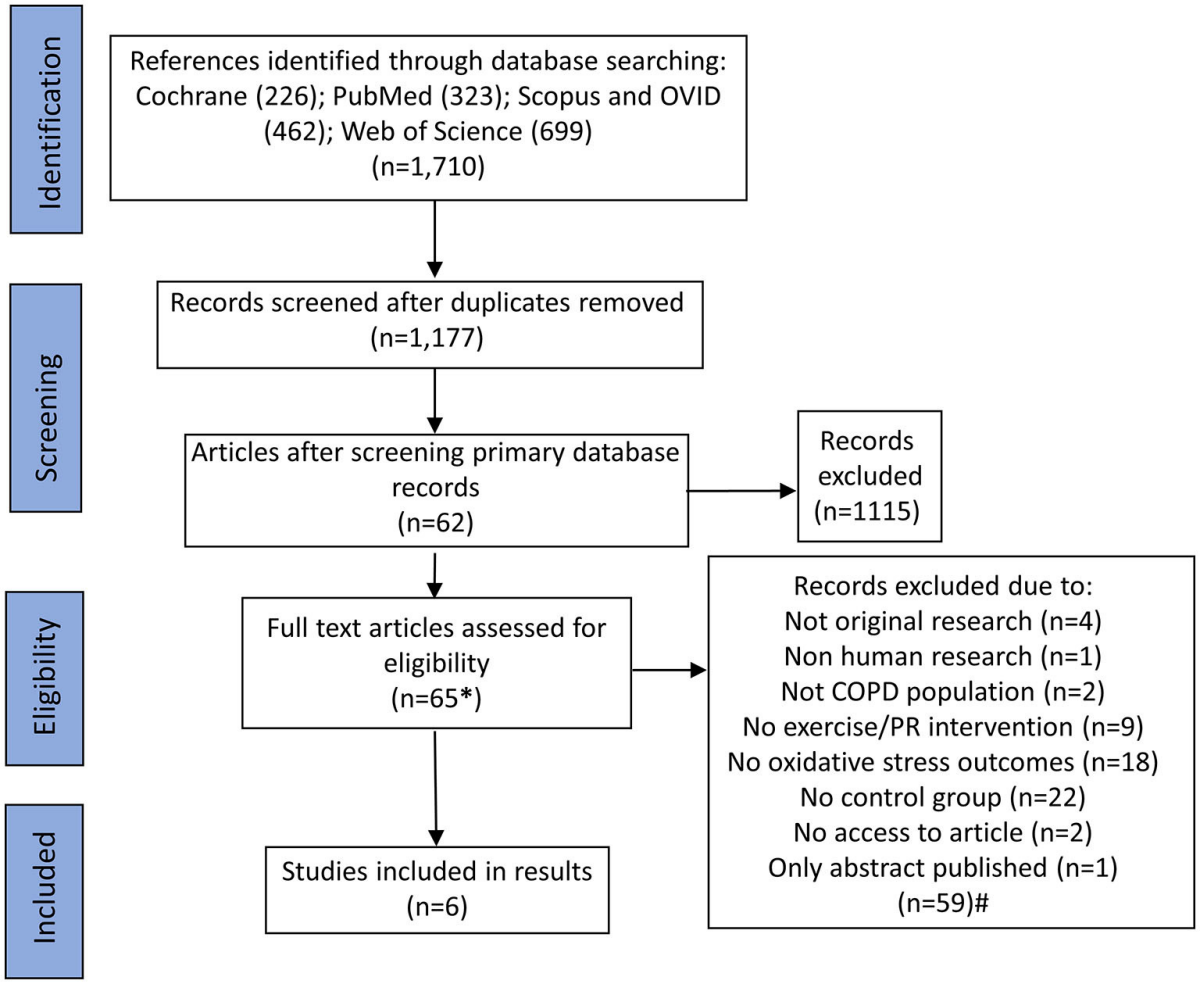
Flowchart of search results and study selection. *An additional three articles were identified through searching Google Scholar, which resulted in 33,900 results being returned; titles and abstracts were screened for the first 100 pages (ordered by relevance; *n* = 1,000). ^#^Some studies had multiple reasons for exclusion. For these studies, the first identified reason for exclusion was used to categorize why that article was excluded to form the n numbers. For articles, which were not able to be accessed, first and corresponding authors were contacted to gain access prior to exclusion.

The search results and selection process are shown in Figure 1. We identifed 1,710 records. After removal of duplicates, 1,117 records were screened by title and abstract and 62 records were identifed for screening of full texts. We further screened the first 1,000 results by title and abstract from Google Scholar, ordered by relevance, and identified an additional three articles. 65 full-text manuscripts were, therefore, screened in full and 59 full-text manuscripts were excluded for various reasons including: not being original research; not being human reseach; not containing a relevant COPD population; not including a relevant exercise or PR intervention; not having relevant outcomes related to redox status; not including a relevant control group; or not having access to the full article or only an abstract being published. Six studies met the relevent inclusion criteria and were selected to be included in this systematic review. Numerous relevant observational studies were identified with appropriate exercise interventions and outcomes. However, these did not include a relevant control group and only reported outcomes before and after exercise in a single COPD group. These, were therefore excluded but are discussed within the discussion. One relevant observational study reported changes in redox status in participants split according to self-reported exercise or no exercise, but no formal PR or exercise intervention was included; this was therefore not included, but again is discussed below (Waseem et al., 2013).

### Characteristics of Studies

The six included studies were small interventional studies with 15–56 participants (8–28 participants in the intervention arm) (Pinho et al., 2007; Chavoshan et al., 2012; Neves et al., 2018; Ryrsø et al., 2018; Tunkamnerdthai et al., 2018; Alcazar et al., 2019). The studies and their characteristics are summarized in Table 4. Four studies were randomized controlled trials (RCTs), one was a non-RCT, and for one it was unclear if there was randomization. The studies mainly recruited mild-to-moderate patients with COPD; Ryrsø et al. (2018) recruited moderate-to-severe patients with COPD with extensive respiratory symptoms. Four studies did not report smoking status or pack year history, one study recruited just ex-smokers (Pinho et al., 2007), and the other study recruited both smokers and ex-smokers (Neves et al., 2018). Patients were clinically stable in four studies and two studies did not state clinical stability. Three studies did not report baseline physical activity levels (Pinho et al., 2007; Chavoshan et al., 2012; Alcazar et al., 2019) and the other three studies reported recruitment of patients performing no regular exercise (Neves et al., 2018; Tunkamnerdthai et al., 2018) or having limited exercise tolerance (Ryrsø et al., 2018). Ages of participants ranged considerably between 45 and 80 years, with some studies having broad age ranges and some narrow age ranges.

**Table 4:** Results of systematic review and included studies.

**Study**	**Study type**	**Population**	**Participants (I vs. C)**	**Intervention and control and time of outcome**	**Outcome**	**Results (Intervention vs. Control)**	**Evidence quality**
Alcazar et al. (2019)	RCT	Stable COPD outpatients ≥65 years old FEV1 47.4 ± 18.1 (I) vs. 58.7 ± 15.2 (C) Baseline activity level not reported but excluded if participated in endurance and/or resistance training programme within previous 12 months Smoking status or pack year history not stated	29 (14 vs. 15)	12-week exercise supervised training (2 sessions/week) programme combining HIIT and power training vs. control of receiving no intervention	Plasma protein carbonylation levels	Decrease in plasma protein carbonyls(−26.9%; *p* <0.05) in I group but no changes in C group (+25.3%; *p* > 0.05)	HIGH risk of bias
Chavoshan et al. (2012)	RCT	Stable COPD patients 55–80 years old FEV1 ≤60%, FEV1/FVC ≤60% Baseline activity level not reported Smoking status or pack year history not stated	40 (8 vs. 11; 22 randomized with additional supplement)	10-week exercise trainer supervised resistance training (3 sessions per week), increasing intensity over time	Skeletal muscle eNOS, nNOS and iNOS protein levels	No significant difference in fold change of eNOS, nNOS or iNOS RNA levelsSignificantly higher fold change of nNOS protein in intervention arm (P <0.05) but no difference in eNOS or iNOS, raw data not provided	HIGH risk of bias
Neves et al. (2018)	Non RCT	Stable COPD patients 45–80 years old FEV1% 58.2 ± 17.2 (I) vs. 58.4 ± 1.4 (C) No regular exercise Current and ex-smokers	20 (10 vs. 10)	12 week supervised whole body vibration training, alternate days, amplitude progressively increasedSampling 1 week before and after exercise intervention	Plasma MDA levels, total antioxidant capacity, SOD activity, catalase activity	No change in MDA protein levels (*P* = 0.22) SOD activity (P=0.74), total antioxidant capacity (*P* = 0.25) or Catalase activity (*P* = 0.95) in either control or intervention group	HIGH risk of bias
Pinho et al. (2007)	Trial, unclear if randomized	COPD patients (Stability not stated) 50–60 years old FEV1 <60% Baseline activity level not reported Ex-smokers	15 (8 vs. 7)	8-week PR In the training program, which included three sessions a week, the subjects cycled at 60% peak maximum oxygen uptake for up to 1 h using cycle ergometer	Plasma total antioxidant capacity MDA Xanthine oxidase activity	Decreased total antioxidant capacity, increased MDA levels, and decreased Xanthine oxidase in Intervention vs. control following intervention (*P* <0.05) Raw data was not provided Total antioxidant capacity only measured after intervention and not measured at baseline	HIGH risk of bias
Ryrsø et al. (2018)	RCT	COPD patients (stability not stated) 63 ± 2 years Moderate to severe COPD (FEV1 26–79%) with extensive respiratory symptoms Limited exercise tolerance Smoking status or pack year history not stated	30 (15 vs. 15)	8 weeks ET, which included 35 min supervised sessions 3 x week of cycling or treadmill walking at moderate intensity to BORG score 14/15 8 weeks RT, which included 4 strength exercises of the major upper and lower body muscle groups (chest press, rowing, leg press, and leg extension); 4 sets with duration of 30 seconds with a 20-second break between sets and a 60-second break between exercises; Load was 3%–40% of one repetition maximum (1 RM)	Skeletal muscle NOX and SOD2	No change in NOX protein levels in either ET or RT SOD2 protein levels increased following both ET and RT to a similar extent (by 53% and 32%, respectively	Risk of bias UNCLEAR
Tunkamnerdthai et al. (2018)	RCT	Clinically stable COPD patients 45–75 years FEV1/FVC <70%, FEV1 30% Not engaging in regular exercise (<3 times/week-1 and <30 min·d-1) Smoking status or pack year history not stated	56 (28 vs. 28)	12 weeks, supervised modified arm swing exercise, 30 min·d-1, 6 d·wk-1 for 12 weeks vs a control group with no intervention but who were “asked to continue with their usual physical activities during the study period”	Plasma MDA levels, Plasma SOD levels	MDA: 3.72 ± 1.46 vs. 2.59 ± 1.01 μM/L; *P* <0.01SOD: (0.95 ± 0.46 vs. 1.44 ± 0.54 U/mL; *P* <0.01	Risk of bias UNCLEAR

*Abstract is only available despite contacting authors.

*I, intervention; C, control; ET, endurance training; RT, resistance training; MDA, malondialdehyde; SOD, superoxide dismutase; NOX, NADPH oxidase*.

Studies used varying supervised 8–12-week exercise regimes, five of which looked at single PR or exercise interventions compared to no intervention, ranging from standard PR to combined high intensity interval training (HIIT) and power training, resistance training, full body vibration training, or modified arm swing (Pinho et al., 2007; Chavoshan et al., 2012; Neves et al., 2018; Tunkamnerdthai et al., 2018; Alcazar et al., 2019) (Table 4). One study compared endurance training with resistance training, but did not include a control without exercise (Ryrsø et al., 2018).

Four studies looked at various relevant systemic measures of redox status through analysis of plasma protein carbonylation levels, malondialdehyde (MDA), superoxide dismutase (SOD) activity, catalase, plasma total antioxidant capacity, lipid peroxidation, and xanthine oxidase activity (Pinho et al., 2007; Neves et al., 2018; Tunkamnerdthai et al., 2018; Alcazar et al., 2019). Two studies looked at only local muscular redox status including endothelial nitric oxide synthase (eNOS), neuronal nitric oxide synthase (nNOS), and inducible nitric oxide synthase (iNOS) RNA and protein levels, NADPH oxidase (NOX), and SOD2 (Chavoshan et al., 2012; Ryrsø et al., 2018).

### Quality of Evidence and Influencing Factors

The evidence available for inclusion in this systematic review was of low quality; overall, four studies had high risk of bias; risk of bias was unclear for the additional two studies (Table 4; Supplementary Table 1). Key problems in the evidence base included availability of only small clinical trials, some of which lacked randomization and only two studies gave details of a randomization process (Neves et al., 2018; Alcazar et al., 2019). None of the RCTs information about concealment. Three studies did not provide details of blinding (Ryrsø et al., 2018; Alcazar et al., 2019), one study was unblinded (Chavoshan et al., 2012), and two studies included single-blinded assessment (Neves et al., 2018; Tunkamnerdthai et al., 2018).

Problems were also found with reporting. There was no mention of intention to treat analysis in any of the studies (Supplementary Table 1). Three studies did not report number of patient dropouts or completeness of outcome reporting; the study by Alcazar et al. (2019) reported a dropout of 35% in the intervention arm vs. 6% in the control arm (Alcazar et al., 2019), the study by Neves et al. (2018) saw a 20% dropout in the intervention arm and 30% dropout of the control arm, the study by Ryrsø et al. (2018) saw a 20% dropout rate in the resistance training group, and no dropouts were mentioned in the endurance training group. However, there was no comment in these studies on differences in baseline characteristics between subjects who completed the study or dropped out and the impact of variables confounding the studies could, therefore, not be excluded. Similarly, the risk of selective outcome reporting was hard to assess for all the studies as no protocol was provided or published.

### Study Outcomes

Some studies did, however, report significant differences in redox status outcomes between intervention and control groups (Table 4). Alcazar et al. (2019) saw a decrease in plasma protein carbonyls, the most abundant byproduct of oxidative-induced protein damage, in the intervention group following the combined HIIT and power training program (−26.9%; *p* < 0.05), but saw no changes in the control group with no intervention (+25.3%; *p* > 0.05) (Alcazar et al., 2019). Tunkamnerdthai et al. (2018) saw significant reductions in MDA levels, a marker of lipid peroxidation, by 1.12 ± 1.18 μM/l (*p* < 0.01) following 12 weeks modified arm swing exercise training, but no change in the control group (−0.38 μM/l, SD could not be calculated; *p* = 0.476) (Tunkamnerdthai et al., 2018). They further saw a significant increase in SOD, an antioxidant enzyme, which has been found to be lower in COPD, by 0.49 ± 0.45 U/ml (*p* < 0.01), but not in the control group (0.08 U/ml, SD could not be calculated, *p* = 0.476). However, Neves et al. (2018) implemented a 12-week whole-body vibration training intervention and saw no change in MDA protein levels (*p* = 0.22), SOD activity (*p* = 0.74), total antioxidant capacity (*p* = 0.25), or catalase activity (*p* = 0.95) in either the intervention or control group. Furthermore, Pinho et al. (2007) did not measure plasma total antioxidant capacity at baseline, but saw lower levels after PR in the intervention group vs. control group (*p* < 0.05). They further saw increased MDA levels (*p* < 0.05) and decreased xanthine oxidase activity (*p* < 0.05) in the intervention group vs. control group.

Two studies looked at the impact of exercise on local muscle redox status, but did not include any measures of systemic redox status (Table 4). The study by Ryrsø et al. (2018) compared endurance training and resistance training and found no change in NOX protein levels in either of the interventions (Ryrsø et al., 2018). However, they saw a similar increase in SOD2 protein levels in both the Endurance training (ET) and Resistance training (RT) (by 53 and 32%, respectively). Comparatively, the study by Chavoshan et al. (2012) found no differences in fold changes of nNOS, eNOS, or iNOS at the RNA level between intervention and control groups, but saw a significantly higher fold increase in nNOS protein levels, but no increase in eNOS or iNOS protein levels, in the intervention arm (*p* < 0.05); raw data were not provided (Chavoshan et al., 2012).

## Discussion

Pulmonary rehabilitation is an essential part of COPD standard of care (Steiner and Roberts, 2016; Bourne et al., 2017; Crooks et al., 2020). However, current guidance does not specify the recommended duration and intensity, and the mechanisms which led to clinical improvements are not well understood. Oxidative stress is a major factor in the pathogenesis of COPD and may be improved by exercise. Understanding which types of exercise intervention will most improve resilience to oxidative stressors is incomplete. Understanding the mechanisms behind this would allow improved targeting of interventions for optimal responses. In this study, we systematically identified and appraised the current evidence around the impact of PR and exercise interventions on redox status. We highlighted the sparsity of high-quality mechanistic evidence. Appropriately designed, high-quality, cross-comparable, and standardized clinical trials are now required and may help to understand the impact of exercise in oxidative stress and pathological COPD mechanisms and the most appropriate exercise intervention for their modification.

We demonstrate that the evidence around the impact of exercise interventions in redox status was sparse and of low quality. Few studies were controlled and those that were controlled had different outcomes and were at either a high risk of bias or the risk of bias was unclear. Furthermore, there was no standardization of trial design. The exercise “dose” was not comparable across groups and many studies were not metabolically controlled to provide equivalent metabolic stress across all the participants (Pinho et al., 2007; Chavoshan et al., 2012; Ryrsø et al., 2018; Tunkamnerdthai et al., 2018; Alcazar et al., 2019). In general, exercise is prescribed by using the Frequency, Intensity, Time, and Type (FITT) principle (Garber et al., 2011), although some argue that this does not incorporate the supervision process and cognitive behavioral domain involved in exercise responses (Ranasinghe et al., 2019) and yet this principle was not referred to in the methods of studies included. Additionally, studies included in this systematic review used exercise doses of varying types and strengths and, therefore, it was impossible to compare outcomes across studies in the same way that it would be impossible to directly compare outcomes of studies by using varying doses of different medications sitting within the same broad medication group (Pinho et al., 2007; Chavoshan et al., 2012; Neves et al., 2018; Ryrsø et al., 2018; Tunkamnerdthai et al., 2018; Alcazar et al., 2019). Length of intervention also varied between 8 and 12 weeks, with additional variation in frequency of exercise per week (Pinho et al., 2007; Chavoshan et al., 2012; Neves et al., 2018; Ryrsø et al., 2018; Tunkamnerdthai et al., 2018; Alcazar et al., 2019). While there are clear guidelines recommending 150 min of moderate-intensity aerobic activity or 75 min of vigorous-intensity aerobic activity and at least 2 days of muscle-strengthening activities per week (Garber et al., 2011), duration of exercise intervention periods is less clear. The 8–12-week time frame is likely reflective of the average duration of pulmonary rehabilitation courses and of exercise interventions demonstrating disease-modifying benefit in other diseases (West et al., 2019). The patient groups themselves were also poorly described; differences in smoking status and previous exercise levels were not well documented and both have been shown to impact on redox status (Hackett et al., 2010; Jamurtas et al., 2018). Similarly, age-related changes in redox status have been demonstrated and previously reviewed (Yap et al., 2009); however, the age range of participants in the studies reviewed herein was broad and not considered in discussions.

The most common exercise interventions investigated were aerobic training with or without resistance training, which is also what tends to be used in PR (Spruit et al., 2013), and have been shown to impact on redox balance in health, as previously discussed (Margaritelis et al., 2020). Two studies included in this review utilized more experimental forms of exercise. Neves et al. (2018) used whole-body vibration as an exercise intervention and investigated the impact of this on MDA levels, total antioxidant capacity, or catalase levels. “Dose” response differences to varying intensities of exercise treatment are a well embedded concept in exercise medicine (Herold et al., 2019) and it may be that whole-body vibration does not provide a great enough “dose” of exercise to impact on redox status. This may well explain the conflicting findings from another intervention study included in this study, which did show change in MDA levels (Tunkamnerdthai et al., 2018) and protein carbonyls (Alcazar et al., 2019). Another consideration is the compartment from which redox status was measured. Most studies sampled blood, which provides a whole body, extracellular picture of redox balance. However, the balance between compartments is a key concept in the redox metabolome (Cortese-Krott et al., 2017) and it may be that differential responses are seen within specific organ compartments. A published abstract by Kelemen et al. (Kelemen et al., 2016) addressed this to an extent, looking at MDA levels in sputum, as a marker of local redox status in the respiratory system and in blood. They saw lower MDA levels in the intervention group following PR compared with the control group (93 ± 20 vs. 145 ± 51 nmol/l) and this difference remained 3 months after PR (102 ± 39 vs. 127 ± 23 nmol/l) (Kelemen et al., 2016). However, this was not included in our analysis, as it was only published as an abstract rather than a full-text article. None of the full-text studies included in our analysis used this approach. Two studies included within our analysis used muscle as their sampling compartment, but without comparison with blood as a surrogate for whole-body redox status (Chavoshan et al., 2012; Ryrsø et al., 2018). Redox changes within muscle may present a more complex picture, as there will be not only the longer term, systemic changes in redox status, but also redox change in response to muscle damage and hypertrophy in response to the exercise intervention. Additionally, consideration is the impact of sarcopenia on redox balance in COPD, with sarcopenia known to impact on the oxidative capacity of skeletal muscle (Dziegala et al., 2018) and, therefore, likely to demonstrate different redox responses to oxidative stimuli such as exercise. Furthermore, some studies have also demonstrated an increase in oxidative stress or lack of improvement with exercise training (Pinho et al., 2007; Neves et al., 2018) with the suggestion that this might be a consequence of overtraining due to the inability of the subjects to adapt to the received exercise dose (Alcazar et al., 2019). This highlights the need for metabolically controlled exercise prescription in COPD that accounts not only for peak exercise capacity of an individual, but also their lactate threshold. The redox metabolites investigated also varied between studies, with the reactive species interactome, a recently conceptualized term to describe these systems, comprising complex and interacting system of metabolites (Cortese-Krott et al., 2017). Sampling of a selected group of redox status markers in a single compartment may not provide an accurate picture of the complex responses to exercise across the body as a whole.

**Figure 2:**
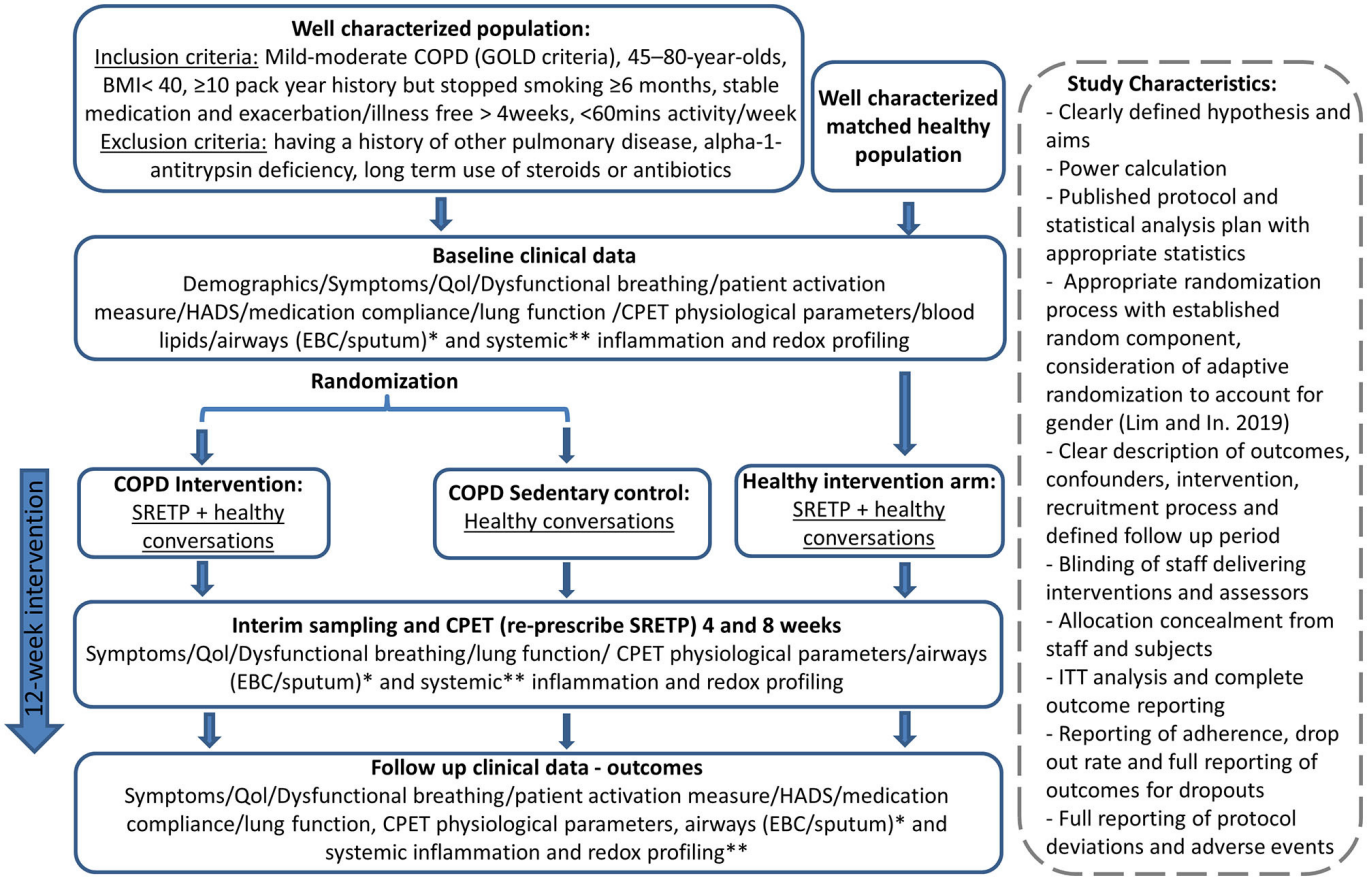
Potential randomized controlled trial (RCT) design to gain high-quality evidence about the impact of exercise on redox status in chronic obstructive pulmonary disease (COPD). The intervention and “dose” were pragmatically chosen, as it has previously demonstrated disease-modulating properties in cancer (West et al., 2019). This is a 8-week twice-weekly supervised structured responsive static cycle-based exercise training programme based on a published protocol (Loughney et al., 2016). This initial study would pave the way for a subsequent study to titrate the dose down to look at the minimal effective dose. Subsequent studies could also include subcohorts of patients on long-term steroids and antibiotics. Healthy conversations will be provided for both the control and exercise intervention group to mitigate for the positive effects of study participation. Healthy conversations are a brief behavior change support intervention, designed to support health behavior change by engaging and motivating clients during brief consultations delivered by practitioners who have received training in Healthy Conversation Skills (HCS). CPET, cardiopulmonary exercise test; EBC, exhaled breath condensate; HADS, hospital anxiety and depression questionnaire; QoL, quality of life; SRETP, structured responsive exercise training program. *Sputum levels of TBARS (lipid peroxidation), total antioxidant capacity, nitrite/nitrate/other nitrosospecies (RXNO) metabolism, and thiol metabolome. **Plasma of: protein carbonylation, malondialdehyde (MDA), superoxide dismutase (SOD) activity, catalase, total antioxidant capacity, TBARS (lipid peroxidation) and xanthine oxidase activity, nitrite/nitrate/other nitrosospecies (RXNO) metabolism, and thiol metabolome.

Adequately blinding and controlling exercise intervention studies are difficult. It is impossible to blind participants to whether they are exercising or not. Similarly, randomized controlled studies, as evidenced by the paucity of studies meeting inclusion criteria for this study, highlight the difficulty in adequately controlling exercise intervention studies. While a matched control group can be used, it is impossible to fully control for the positive impact of study participation in the exercise group, who may have supervised exercise sessions two to three times per week, with all the associated benefit of regular contact with a healthcare professional. Further to this, the control group is likely to be motivated individuals by default and, therefore, may initiate additional physical activity following enrolment into the control rather than intervention group, which could then dilute the perceived impact of exercise intervention in the results. A number of studies that did not meet the inclusion criteria for this review attempted to circumvent this by using participants as their own internal controls. Baseline and post-intervention results of the participants were compared rather than comparing an intervention and control groups. Potential for confounding also exists with this study design; study participation may result in increased compliance with medication, and, as with RCTs, there is additionally the positive impact of study participation to consider. An observational study by Waseem et al. (2013) demonstrated improvements in a number of redox parameters, including SOD, catalase, MDA, and glutathione peroxidase (GPX), but as with the RCTs identified in this systematic review, evidence quality was poor when we assessed it by using the modified Downs and Black checklist (Downs, 1998). It is important to note that, although the methodology was developed prior to undertaking this systematic review, no formal protocol was written and it was not registered, for example, on PROSPERO (National Institute for Health Research, 2021). Use of a formalized published protocol will be useful for a future systematic review on emerging evidence. However, we have demonstrated that overall there is a lack of high-quality evidence and that well-designed standardized studies are now required. We have described an idealized RCT design, which could be used to try and meet this need and this is given in Figure 2.

In summary, this systematic review, aiming to understand the impact of different intensities and types of exercise intervention on the redox metabolome and clinical outcomes in COPD, has demonstrated a lack of high-quality evidence in this field. While exercise interventions in COPD are known to improve outcome and appear to affect the redox capacity of patients with COPD, the most notable finding from this study is that further work is needed in this area. This future work is essential as a greater understanding of the mechanism through which exercise confers improvement in COPD allows opportunity for greater personalization of exercise intervention programs and potential for identification of new drug targets.

## Author Contributions

AW and AF developed the search terms and search strategy, performed the literature search, reviewed the search results, extracted the data, appraised the studies, administered the project, and wrote the initial draft. TW supervised the project. AW, TW, and AF conceived the project, reviewed and edited the manuscript, and approved the final draft. All authors contributed to the article and approved the submitted version.

## Conflict of Interest

TW reports being a founder and director of, and a shareholder in my mhealth; receiving research grants for trials of interferon beta and other COVID-19 treatments from AstraZeneca, GlaxoSmithKline, Synairgen, Bergenbio, UCB, National Institute for Health Research (NIHR), UK Research and Innovation (UKRI), and my mhealth; receiving consultancy fees from AstraZeneca, Synairgen, my mhealth, Valneva, OM Pharma, Boehringer Ingelheim, and Roche; receiving fees for attending lectures and meetings from Boehringer Ingelheim, AstraZeneca, Chiesi, Teva, and GlaxoSmithKline; receiving travel support for attending conferences and meetings from Nutricia, AstraZeneca, Chiesi, Boehringer Ingelheim, and GlaxoSmithKline; applying for patents for bacterial vaccines with GlaxoSmithKline and my mhealth; being a member a specialist chronic obstructive pulmonary disease advisory group; and being a member of the independent data monitoring committee (IDMC) of a vaccine study sponsored by Valneva and Synairgen. These are all outside the submitted work. The remaining authors declare that the research was conducted in the absence of any commercial or financial relationships that could be construed as a potential conflict of interest.

## Publisher's Note

All claims expressed in this article are solely those of the authors and do not necessarily represent those of their affiliated organizations, or those of the publisher, the editors and the reviewers. Any product that may be evaluated in this article, or claim that may be made by its manufacturer, is not guaranteed or endorsed by the publisher.
