# Supplementary material for: Evidence Around the Impact of Pulmonary Rehabilitation and Exercise on Redox Status in COPD: A Systematic Review
Source: Front Sports Act Living. 2021 Nov 26;3:782590. doi: 10.3389/fspor.2021.782590 (PMC8664411; doi:10.3389/fspor.2021.782590)
Supplement: Supplementary file 1 [file Table_1.DOCX]

Supplementary Material

# Supplementary Tables

**Supplementary Table 1. Results from studies using simplified ROB2 tool to assess evidence quality**

| **Author year** | **Sequence generation** | **Allocation concealment** | **Blinding** | **Incomplete outcome reporting** | **Selective outcome reporting** | **Other** |
| --- | --- | --- | --- | --- | --- | --- |
| Alcazar et al. 2019 (1) | LOW  Stratified randomisation was used to achieve approximate balance regarding physical function (SPPB < 10 and SPPB ≥10) and sex (women and men) | UNCLEAR no mention of allocation concealment | HIGH  No mention of blinding but appears no blinding was used | HIGH  No mention of ITT analysis  35% (Exercise) vs 6% Control dropped out  No comment on the baseline characteristics of dropouts vs. completers. But reasons for drop out explained | UNCLEAR  No Protocol | Baseline data provided for all participants. But small trial and lung function differences are present, but small n numbers may mean that they are not significant |
| Chavoshan et al. 2012 (2) | UNCLEAR | UNCLEAR | HIGH  The exercise intervention part of trial was Unblinded | UNCLEAR  No mention of ITT analysis. Not clear how many dropouts for each group | UNCLEAR  No Protocol | 13 randomized subjects not providing biopsies had lower FEV1 and higher, but did not differ significantly in other characteristics. Differences in dropouts between groups not discussed |
| Neves et al. 2018 (3) | HIGH  No randomisation | HIGH  Unconcealed | UNCLEAR  Single-blinded assessment, patients not blinded | UNCLEAR  2 dropped out of intervention-lack of motivation may introduce bias. 3 dropped out of control arm-acute exacerbation, moved out of area and 1 had initial physical treatment (?). Flow through trial displayed in figure | UNCLEAR  no protocol | Non significant differences between baseline parameters |
| Pinho et al. 2007 (4) | UNCLEAR | UNCLEAR | HIGH  No blinding | UNCLEAR | UNCLEAR  no protocol | Unclear study, hard to interpret methodology and results. Total antioxidant capacity and other parameters not measured at baseline. |
| Ryrsø et al. 2018 (5) | UNCLEAR | UNCLEAR | UNCLEAR | HIGH  2 (13%) patients lost to follow up in resistance training group due to exacerbation and neck pain | UNCLEAR no protocol | Small study, no COPD sedentary controls |
| Tunkamnerdthai et al. 2018 (6) | UNCLEAR  Randomization process not stated | UNCLEAR | HIGH  single-blinded assessment, no further details provided | HIGH  3 (30%) dropped out of the control arm and 2 (20%) from the intervention arm. ITT analysis not mentioned but unlikely. Flow through trial displayed in protocol figure | UNCLEAR no protocol | Baseline parameters look the same |

ITT = Intention to treat analysis

Results were generated using a simplified version of the ROB2 tool (7).

*The overall bias rating was defined by the highest single risk of bias category assessed for the paper or if parameters were unclear then the overall rating was defined as unclear.*

1. Alcazar J, Losa-Reyna J, Rodriguez-Lopez C, Navarro-Cruz R, Alfaro-Acha A, Ara I, et al. Effects of concurrent exercise training on muscle dysfunction and systemic oxidative stress in older people with COPD. Scand J Med Sci Sports. 2019;29(10):1591-603.

2. Chavoshan B, Fournier M, Lewis MI, Porszasz J, Storer TW, Da X, et al. Testosterone and resistance training effects on muscle nitric oxide synthase isoforms in COPD men. Respiratory medicine. 2012;106(2):269‐75.

3. Neves CDC, Lacerda ACR, Lage VKS, Soares AA, Chaves MGA, Lima LP, et al. Whole body vibration training increases physical measures and quality of life without altering inflammatory-oxidative biomarkers in patients with moderate COPD. Journal of Applied Physiology. 2018;125(2):520-8.

4. Pinho RA, Chiesa D, Mezzomo KM, Andrades ME, Bonatto F, Gelain D, et al. Oxidative stress in chronic obstructive pulmonary disease patients submitted to a rehabilitation program. Respir Med. 2007;101(8):1830-5.

5. Ryrsø CK, Thaning P, Siebenmann C, Lundby C, Lange P, Pedersen BK, et al. Effect of endurance versus resistance training on local muscle and systemic inflammation and oxidative stress in COPD. Scandinavian journal of medicine & science in sports. 2018;28(11):2339‐48.

6. Tunkamnerdthai O, Auvichayapat P, Punjaruk W, Manimmanakorn A, Leelayuwat N, Boonsawat W, et al. Modified Arm Swing Exercise Improves Oxidative Stress and Heart Rate Variability in Patients with Chronic Obstructive Pulmonary Disease: A Randomized Controlled Trial. Journal of Exercise Physiology Online. 2018;21(4).

7. Cochrane Methods Bias. 2020. RoB 2: A revised Cochrane risk-of-bias tool for randomized trials. Available at <https://methods.cochrane.org/bias/resources/rob-2-revised-cochrane-risk-bias-tool-randomized-trials> [Accessed 17th August 2021].
